# Association of Air Quality Improvement and Frailty Progression: A National Study across China

**DOI:** 10.3390/toxics12070464

**Published:** 2024-06-27

**Authors:** Mingqin Liu, Mohammed Zeeshan, Tiantian Sun, Xiangming Hu, Zhiqiang Nie, Haojian Dong, Guanghui Dong, Yanqiu Ou

**Affiliations:** 1 Guangdong Cardiovascular Institute, Guangdong Provincial People’s Hospital, Guangdong Academy of Medical Sciences, Guangzhou 510080, China; liumingqin@gdph.org.cn; 2 Department of Biochemistry and Structural Biology, University of Texas Health San Antonio, San Antonio, TX 78229, USA; mdzeesh@gmail.com; 3 Department of Hematology, The Seventh Affiliated Hospital, Sun Yat-sen University, Shenzhen 518000, China; suntt6@mail.sysu.edu.cn; 4 Department of Cardiology, Guangdong Provincial People’s Hospital, Guangdong Academy of Medical Sciences, Southern Medical University, Guangzhou 510080, China; hxmgdcz@163.com (X.H.); niezhiqiang@gdph.org.cn (Z.N.); donghaojian@gdph.org.cn (H.D.); 5 Guangdong Provincial Engineering Technology Research Center of Environmental Pollution and Health Risk Assessment, Department of Occupational and Environmental Health, School of Public Health, Sun Yat-sen University, Guangzhou 510080, China

**Keywords:** air quality improvement, PM_2.5_, ageing, frailty, public health, CHARLS

## Abstract

Accumulating evidence strongly suggests that exposure to ambient air pollution is linked with increased frailty. However, little is known about the effect of improved air quality on frailty progression. We aimed to investigate whether improvements in air quality (PM_1_, PM_2.5_, PM_10_, NO_2_, and O_3_) can alleviate frailty progression, particularly in the aftermath of implementation of the “Clean Air Action” policy in China. The study involved 12,891 participants with geocoded environmental data from the nationwide China Health and Retirement Longitudinal Study (CHARLS) during the period from May 2011 to August 2015. Multivariate logistic regression models were used to analyze the association of air pollution improvements and frailty progression. The protective effects were noted for PM_1_, PM_2.5_, PM_10_, and NO_2_ indices, with an aOR (adjusted odds ratio) ranging from 0.72 to 0.79. Air quality improvement in PM_1_, PM_2.5_, PM_10_, and NO_2_ could alleviate the progression of frailty. The study is the first to examine the association between the improvement of air quality and the progression of frailty, setting a precedent for the importance of a nationwide clean air policy and its impact on healthy ageing.

## 1. Introduction

Frailty is a geriatric syndrome that is distinguished by a decline in physiologic reserves and an increase in vulnerability to stressors. This condition is closely linked to a multitude of adverse health outcomes, such as falls, hospitalization, and mortality [1,2]. Frailty, serving as an intermediary phase between healthy aging and disability, is an emerging worldwide health concern with significant ramifications for both clinical practice and public health. Given the ongoing rapid aging of the population, the anticipated surge in frailty prevalence is inevitable. Several studies have demonstrated a clear connection between frailty and increased health-care costs and utilization [3,4,5]. Frailty is dynamic, meaning it may change over time and be reversible [1,6]. There exists a multitude of factors that may contribute to the initiation or advancement of frailty, encompassing sociodemographic, clinical, lifestyle-related, and physiological aspects. Comprehending these risk factors holds significance in formulating public health and preventive measures, particularly when the risk factors are subject to modification. Sociodemographic factors include age, gender, level of education, as well as socioeconomic position and living environment factors like air quality and residential greenness. Clinical factors include genetics, chronic conditions such as diabetes, cardiovascular conditions, as well as the use of medication. Lifestyle-related factors include diet and physical activity, while biological factors encompass markers of inflammation, the endocrine system, and micronutrients. Thus, identification of modifiable risk factors and effective interventions can contribute to the reduction of the occurrence and progression of frailty in the elderly population.

Air pollution is a widespread environmental threat that poses significant risks to public health, particularly in developing nations. Recent epidemiological investigations have revealed a connection between exposure to air pollutants and a heightened vulnerability to frailty [7,8,9]. To illustrate, Guo et al. conducted a study examining the long-term effects of ambient PM_2.5_ exposure on frailty in six low- and middle-income countries. The findings indicate a 30% increase in the likelihood of frailty in rural regions for every 10 μg/m^3^ rise in ambient PM_2.5_ levels. It is worth noting that PM_1_, which is smaller in size than PM_2.5_, not only enters the blood circulation system but can also invade the brain through the olfactory bulb, breaching the blood–brain barrier and affecting the nervous system [10]. Further, PM_1_ can be deposited in deeper parts of the human body and may influence the functioning of other organs leading to more serious health problems. Interestingly, there have been no studies on the association between PM_1_ and frailty and its progression. Similarly, an increasing number of studies find that ozone (O_3_) and nitrogen dioxide (NO_2_) are closely related to an increased risk of cardiovascular and metabolic diseases [11,12,13], while study on their relationship with frailty is still less. Studying the influence of various pollutants on the progression of frailty may provide a more comprehensive insight of their relationships.

In recent years, China has implemented various policies to regulate air pollution, leading to a notable enhancement in the air quality across the nation and a substantial decrease in instances of severe pollution. The Chinese government introduced the China Air Pollution Prevention and Control Action Plan (APPCAP), commonly referred to as the “Clean Air Action” policy, in 2013. This comprehensive policy is widely regarded as the most rigorous initiative to date and seeks to ameliorate air quality while mitigating the health risks associated with air pollution. The plan encompasses strategies such as optimizing industrial frameworks, curtailing the utilization of unclean fuels, and advancing clean energy technologies. [14,15]. In 2017, major Chinese cities experienced a significant decrease of 33.3% in PM_2.5_ concentrations compared to 2013, as reported by Huang et al. [16]. Currently, there is a scarcity of research examining the beneficial impacts of the Clean Air Action policy on the progression of frailty.

In this study, using frailty index (FI) to quantify the frailty progression, we aim to evaluate the impact of implementation of the Clean Air Action policy on frailty progression in the public by combining China Health and Retirement Longitudinal Study (CHARLS) data and the air pollution monitoring data.

## 2. Materials and Methods

### 2.1. Study Population

**Figure 1:**
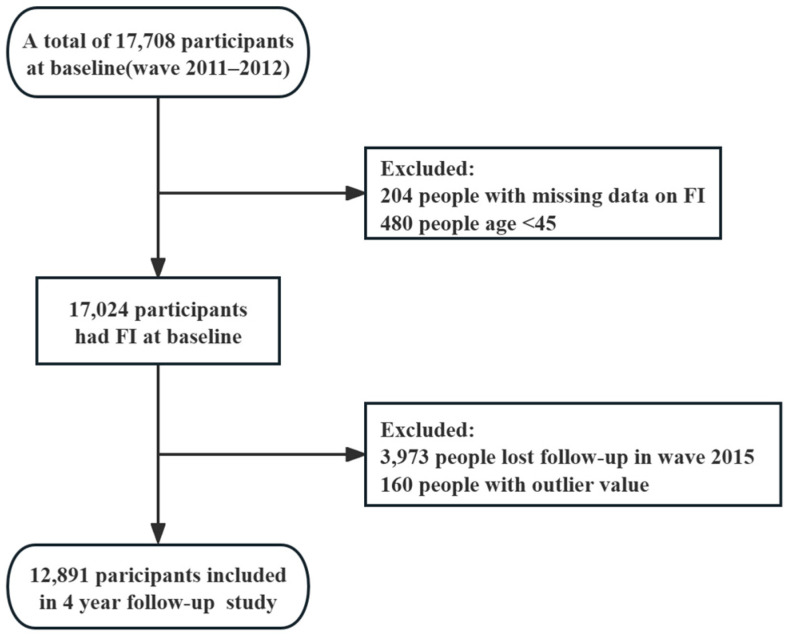
Flow chart of the study population selection.

The present study made use of data collected from wave 1 (May 2011) and wave 3 (August 2015) of the China Health and Retirement Longitudinal Study (CHARLS). This longitudinal study consists of a nationally representative cohort of approximately 17,708 participants residing in 450 urban communities and rural areas throughout China. Zhao et al. have provided a comprehensive description of the study design, including the sampling methods, data acquisition procedures, and data quality assessment [17]. In order to enhance the reliability of our analysis, specific criteria were employed for the selection of participants. This entailed excluding individuals below the age of 45 (*n* = 480), those with incomplete data on FI (*n* = 204), individuals with outlier value (*n* = 160), and those who were lost to follow-up (*n* = 3973). As a result, the final cohort consisted of 12,891 participants, as depicted in Figure 1. The ethical considerations pertaining to our research were appropriately addressed, with the primary study obtaining approval from the ethical committee of Peking University. Furthermore, all participants were required to provide written informed consent prior to their inclusion in the study.

### 2.2. Air Pollution Assessment

Ambient air pollutants (PM_1_, PM_2.5_, PM_10_, NO_2_, and O_3_) in this study were estimated by a satellite-based random forest approach, which has been recorded previously in detail [18,19,20,21,22,23]. Concisely, a random forest model (based on a machine learning algorithm) was used for model development, which incorporated satellite-observed aerosol optical depth and tropospheric NO_2_ from the ozone-monitoring instrument as independent variables, with ground-level concentrations of air pollutants as dependent variables. Additional predictors were also obtained at the model development period, including meteorological factors (e.g., temperature, relative humidity, wind speed, and barometric pressure) and land use data (e.g., percentage of urban cover and greenness). The validated model estimated air pollutant concentrations in the study area at a 0.01° × 0.01° spatial resolution (≈1 km). Personalized daily exposure was estimated by linking the residential address of participants, and these daily exposures were then aggregated into annual averages. In this study, we define the air quality improvement as follows: Δ air pollutants (e.g., ΔPM_1_, ΔPM_2.5_, ΔPM_10_, ΔO_3_, and ΔNO_2_) level = air pollutants level in 2011 − air pollutants level in 2015.

### 2.3. Frailty Index Assessment

The evaluation of frailty is carried out through the utilization of two widely employed clinical tools, namely the frailty phenotype and frailty index (FI), which are based on the deficit accumulation model. The FI, being a continuous scale measure, exhibits high sensitivity even at the lower range of the frailty spectrum, thereby enabling investigations involving younger individuals [24]. FI was constructed based on methods developed in previous studies [25,26]. It was developed by utilizing 53 items of health deficit data across five dimensions, namely physical limitations, psychological symptoms, comorbidities, history of trauma, and cognitive impairment, as outlined in Supplementary Materials Table S1. FI was operationalized as the summation of reported deficits divided by the total number of possible deficits answered, resulting in a score ranging from 0 to 1. Higher scores on the FI indicate a greater degree of frailty. To determine the absolute change in FI for an individual, the calculation involved subtracting the FI score in 2011 from the FI score in 2015 (ΔFI = FI 2015 − FI 2011). The occurrence of “frailty progression” was identified as a positive difference between the 2011 and 2015 frailty index assessments (ΔFI > 0). No change or negative changes (ΔFI ≤ 0) indicates “ no frailty progression”. In order to ensure the precision of the frailty index, individuals with a denominator less than 42 (80% of 53 entries) were excluded from the study. The participants were assessed utilizing this FI during wave 1 and wave 3.

### 2.4. Covariates

Based on the previous literature and clinical evidence [7,8,9,24,27], covariates in the current analysis included age (continuous), sex (male/female), BMI (continuous), marital status (separated or divorced, married but temporarily separated, and married and living together), educational level (primary school and below, junior high school, high school and above), residence (urban/rural), drinking status (never, <1 time per month, and >1 time per month), smoking (yes/no), sleeping time (continuous), insurance (yes/no), cooking fuel (clean fuel/solid fuel), and social activity (yes/no).

### 2.5. Statistical Analyses

The study population’s baseline characteristics were reported using numbers and percentages for categorical variables and means and standard deviations for continuous variables. A *t*-test was employed to analyze continuous variables, while a χ^2^ test was used for categorical variables. To investigate the correlation between changes in air pollutants, Spearman rank correlation analysis was conducted. The odds ratio (OR) and a 95% CI (confidence interval) for the association of air quality improvement and frailty progression were calculated using a multivariate logistic regression model. Model 1, referred to as the crude model, was utilized for the initial analysis. In the subsequent multivariable logistic regression, confounders were selected based on two criteria: (1) if they resulted in a change of the effect estimate for the association between air pollution and frailty progression by more than 10%; and (2) if they were significantly associated with both air pollution and frailty progression. Model 2 included the confounders of age, sex, and BMI. Building upon Model 2, Model 3 further incorporated additional confounders, including residence, educational level, marital status, smoking, insurance, drinking status, sleeping time per day, and social activity. Recognizing the potential influence of baseline FI on the progression of frailty, Model 4 further accounted for baseline FI. In order to investigate the potential non-linear association between improvement in air quality and frailty progression, restricted cubic splines (RCS) were employed. The reference value (OR = 1) was established at the 10th percentile, and the knots were placed at the 10th, 50th, and 90th percentiles of the ln-transformed concentrations. Values falling outside the 5th and 95th percentiles were excluded. Additionally, the Bayesian kernel machine regression (BKMR) model was employed to assess the collective impact of various air pollutants, taking into account their potential nonlinearity and combined effects. To conduct subgroup analyses, the samples were stratified by various factors, including age (≥65 years), sex, BMI, residence, smoking, and cooking fuel. Sensitivity analysis was conducted as follows: (1) using binary classification of frailty progression by dichotomizing frailty progression at the 75th percentile of ΔFI distribution—frailty progression was defined as ΔFI ≥ 0.06990; (2) using Δ ambient air pollutants (e.g., ΔPM_1_, ΔPM_2.5_, ΔPM_10_, ΔO_3_, and ΔNO_2_) level as a continuing variable. Data analysis was carried out using SPSS 26.0 and R software (Version 4.3.1). To determine the statistical significance of the data, a two-tailed *p*-value < 0.05 was calculated.

## 3. Results

### 3.1. Baseline Characteristics

The study included a total of 12,891 participants, comprising 6240 men and 6651 women (Table 1). The participants had a mean age of 58.6. Out of the total participants, 6810 experienced frailty progression. It is observed that participants who have frailty progression are more likely to be female, reside in rural areas, have lower educational levels, lower rates of smoking and drinking, and higher FI at baseline compared to those without frailty progression. Table 2 provides information on the average concentrations and changes in air pollutants from 2011 to 2015. The greatest improvement was observed in PM_1_, PM_10_, and O_3_. Furthermore, significant differences were found in the changes (∆) of PM_1_ (0.79 vs. 1.07 μg/m^3^, *p* = 0.002), PM_2.5_ (−1.20 vs. −1.03 μg/m^3^, *p* = 0.008), PM_10_ (12.27 vs. 14.51 μg/m^3^, *p* < 0.001), and NO_2_ (−1.53 vs. −1.34 μg/m^3^, *p* = 0.010) between individuals with and without frailty progression.

### 3.2. Air Quality Improvement and Frailty Progression

The changes of FI in different quartiles of pollutant quality improvement from 2011 to 2015 by different pollutants are shown in Figure S1, in which we observed smaller progression of FI with greater improvement of the air quality by different pollutant indices. This study presents the associations between various levels of exposure to ambient air pollution and the progression of frailty, as depicted in Table 3. For PM_1_, after fully adjusting for covariates, the aOR for Q4 compared to Q1 of PM_1_ reduction in Model 4 was 0.75 (0.68~0.84). For PM_2.5_, compared to Q1, Q2 to Q4 improvement was inversely associated with the risk for frailty progression [aOR for Q4 = 0.72 (0.65~0.80)], and showed a greater protective trend from Q2 to Q4 (*p* for trend < 0.01). There were similar protective effects of air quality improvement on frailty progression for PM_10_ and NO_2_. The aOR for the Q4 compared to the Q1 of air quality improvement was 0.73 (95% CI: 0.66–0.81) for PM_10_ and 0.79 (95% CI: 0.71–0.88) for NO_2_, while the reduction in O_3_ level appeared to have a weaker association with changes in frailty progression, as indicated by an aOR of 0.94 (95% CI: 0.85–1.04). In Model 3, the reduction of PM_10_ exhibits a protective role against frailty progression [aOR: 0.80 (0.73~0.88)]. This effect has been seen to a diminished degree in PM_1_, PM_2.5_, and NO_2_, while the decrease in O_3_ may not offer significant protection [aOR: 0.98 (0.89~1.08) in Model 3]. The protective effects and trend of the air pollutants (e.g., PM_1_, PM_2.5_, PM_10_, and NO_2_) on frailty progression tends to be similar in Model 1 and Model 2.

**Figure 2:**
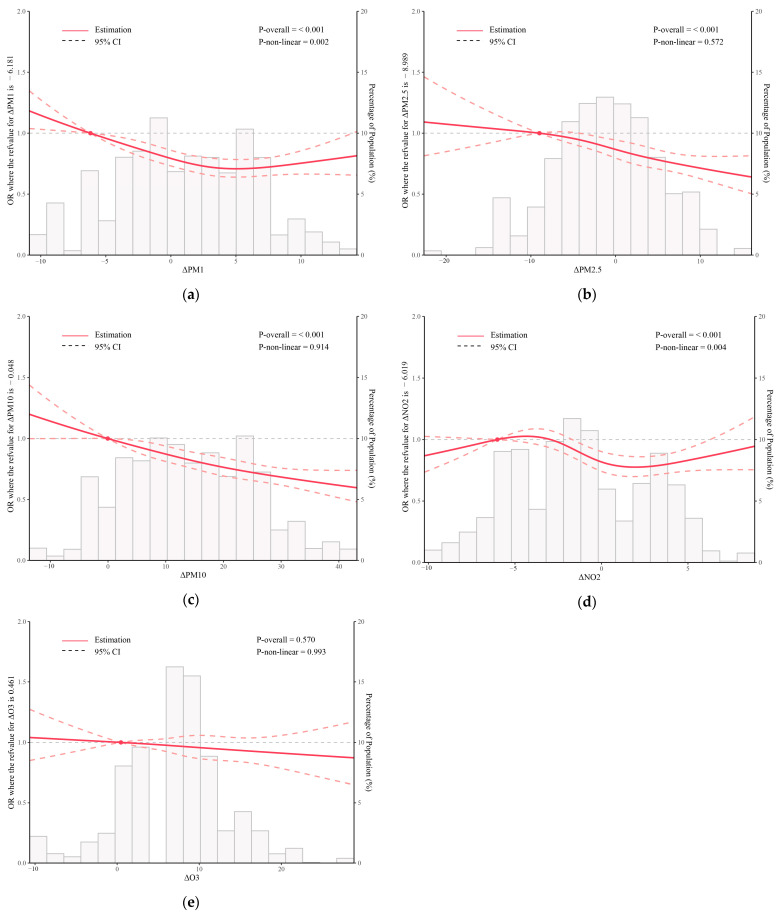
Restricted cubic spline for the associations between changes in air pollutant concentrations and frailty progression: (**a**) ΔPM_1_, (**b**) ΔPM_2.5_, (**c**) ΔPM_10_, (**d**) ΔNO_2_, (**e**) ΔO_3_. The lines represent odds ratios (ORs, solid lines) and 95% confidence intervals (CIs, long dashed lines) after multivariable adjustment for age, sex, and BMI, residence, educational level, marriage status, smoking, insurance, drinking status, sleeping time per day, social activity, and FI at baseline based on the RCS models for the ln-transformed concentrations of air pollutant. The reference values (OR = 1) were set at the 10th percentiles, and the knots were set at the 10th, 50th, and 90th percentiles of the ln-transformed air pollutant concentrations. The histograms represent the distribution of concentrations of air pollutants in our study, excluding values outside the 5th and 95th percentiles. PM_1_: particulate matter with an aerodynamic diameter < 1 μm; PM_2.5_: particulate matter with an aerodynamic diameter < 2.5 μm; PM_10_: particulate matter with an aerodynamic diameter < 10 μm; NO_2_: nitrogen dioxide; O_3_: ozone.

Restricted cubic splines analysis revealed that as the levels of PM_1_, PM_2.5_, and PM_10_ improved, the mitigating effects against frailty progression became more pronounced (Figure 2). The curve showed a linear and negative association between change in PM_2.5_ and the frailty progression (linear *p*-value < 0.001; nonlinear *p*-value = 0.572). Similarly, change in PM_10_ presented an overall linear dose–response trend with frailty progression (linear *p*-value < 0.001; nonlinear *p*-value = 0.914). Furthermore, the correlation between alterations in different air pollutants from 2011 to 2015 was assessed and visualized in Figure S2. There was a stronger positive correlation between alterations in PM_1_ and PM_10_ (ρ = 0.69). And alterations in PM_1_ and PM_2.5_ exhibited a similar positive correlation (ρ = 0.65). Conversely, alterations in O_3_ exhibited the lowest correlation with changes in other air pollutants. To investigate the potential combined effects of the changes in these five different air pollutants on the risk of frailty progression, a BKMR model was employed. When the overall concentrations of changes in five air pollutants (PM_1_, PM_2.5_, PM_10_, NO_2_, and O_3_) are higher than P_70_, the risk of frailty progression decreases as the changes in pollutants concentration increase (Figure S3a). When evaluating the impact of single air pollutant on frailty progression, it was found that most of the results were not statistically significant when other air pollutants were fixed at P_25_, P_50_, and P_75_, respectively. Only when other pollutants are fixed at P_50_ does the risk of frailty progression increase with the improvement of NO_2_ (Figure S3b).

### 3.3. Subgroup and Sensitivity Analysis

Subgroup analyses revealed the reduction in PM_1_ was found to have a more pronounced protective effect for females compared to males [aORs: 0.88 (0.84–0.92) vs. 0.93 (0.90–0.98)]. In addition, the improvement of PM_10_ levels showed a more significant protective effect in individuals who were smokers [aORs: 0.85 (0.81–0.90) vs. 0.93 (0.89–0.97)] and those who used solid fuels for cooking [aORs: 0.87 (0.84–0.91) vs. 0.93 (0.88–0.98)]. Similarly, the protective effects of NO_2_ reduction on frailty progression were also influenced by the use of solid fuels [aORs for solid fuel compared to clean fuel: 0.90 (0.86–0.94) vs. 0.97 (0.92–1.02)]. However, the estimated subgroup-specific aORs for O_3_ were found to be close to 1. (Figure S4).

In sensitivity analysis, the aORs of Model 4 ranged from 0.65 to 0.97, indicating frailty progression associated with a reduction of air pollution (Q4 vs. Q1) (Table S2). Simultaneously in Table S3, when transforming Δambient air pollutants levels into continuing variables, air quality improvements in PM_1_, PM_2.5_, PM_10_, NO_2_ have similar protective effectives on frailty progression, with aORs ranging from 0.98 to 0.99 for per 1 μg/m^3^ increase. These results and trend were all consistent with our major analysis.

## 4. Discussion

In this nationwide cohort study with a median follow-up of 4 years, we investigated the protective effect of air quality improvement on frailty progression and assessed the dose–response relationship between air pollution change and frailty progression. Across all subgroups of the population studied, the association between air quality improvement and frailty benefits for PM_1_, PM_2.5_, PM_10_, and NO_2_ was consistently evident, while the reduction of O_3_ had no significant impact in protecting against frailty progression. To some extent, clean air action has a positive effect in slowing frailty progression. To the best of our knowledge, this study is the first of its kind to explore the effects of improved air quality on frailty progression in the Chinese population aged 45 and over.

Our findings are consistent with previous studies conducted in China, South Korea, and the United Kingdom [9,28,29], which reported protective benefits of improved air quality against frailty. In a cross-sectional study of 2912 elderly people (age ≥ 70 years) in South Korea [9], Shin et al. evaluated the risk of exposure to PM_2.5_, PM_10_, and O_3_ on frailty in different status (robust, pre-frail, and frail) groups. The study confirmed that increased concentrations of PM_2.5_, PM_10_, and O_3_ have been linked to a higher risk of being frail or pre-frail compared to those in the robust group. Because their study is a cross-sectional study, the study cannot verify the causal relationship between frailty and exposure to PM_2.5_, PM_10_, and O_3_, and it also doesn’t assess the impacts of pollutants on the progression of frailty. In our study, we utilized a national cohort to further explore their longitudinal relationships through a 4-year follow-up. We observed a positive effect of reducing PM_2.5_ and PM_10_ levels on the protection against frailty progression. However, the reduction in O_3_ levels did not show a significant effect in this regard. This discrepancy could be attributed to the possibility that levels of ozone improvement did not differ significantly, leading to potential benefits being equally distributed between the with and without frailty progression groups. Additionally, the potential pathophysiological mechanisms of the relationship between O_3_ and frailty were still unclear. An anti-inflammation effect was also reported with O_3_ exposure, in addition to a pro-inflammation effect [30]. On the other hand, O_3_ was more likely to be influenced by lifestyle choices than particulate matters, like level of outdoor physical activity [23,31]. Therefore, further studies controlling for these potential confounders will help to clarify the discrepancy. A cross-sectional study from the UK’s Biobank found that exposure to NOx was associated with a higher risk of being in both the pre-frail and frail categories [29]. Correspondingly, our study also found that reduction in NO_2_ was related to reducing the risk of frailty progression. These findings suggest that reductions in NO_2_, in addition to PM_2.5_ and PM_10_, also contribute to the protective role against frailty progression.

Previous studies had primarily focused on the effects of air pollution and the risk of frailty in individuals who are over the age of 60. However, our study included participants aged 45 and above. Our findings indicated that air pollution reduction alleviated the progression of frailty in those aged 45 and over. This suggests that air quality improvements could play a crucial role in aiding the prevention and management of frailty in a broader population. Although in subgroup analysis the effects of air quality improvement in PM_1_, PM_2.5_, PM_10_, NO_2_, and O_3_ on the progression of frailty were not affected by age, the results were statistically significant for participants aged < 65 years. These results suggest the need for relevant screening and intervention programs in middle-aged people. Additionally, we considered whether air pollution mixtures play a role on frailty progression, for which a BKMR statistical model was conducted. BKMR analysis had advantages over traditional statistical models (logistic or linear regression) in analyzing multi-pollutant exposures. In the mixed multi-pollutants model, when the overall improvement concentration of pollutants is higher than P_70_, the risk of frailty progress decreases as the improvement of pollutant concentration increases. But we did not find significant correlations in the single-pollutant model.

In a review conducted by García-Esquinas et al., the adverse effects of environmental pollutants on frailty in older adults were summarized [32]. The review highlighted that long-term exposure to air pollution not only increases the risk of frailty but also its associated consequences. Frailty is a prevalent condition among older adults, with approximately 15% of older community residents in the United States being classified as frail in 2011 [33]. Similarly, in Europe, data from the Survey of Health, Aging, and Retirement in Europe revealed that in 2014, more than 50% of adults aged 50 and older experienced limitations in mobility and functioning, while approximately 10% were identified as frail [34,35]. As frailty is an increasingly frequent problem among older people, leading to serious health and disability issues, it is essential to identify and address modifiable determinants that can help prevent the progression of frailty. Our study has revealed that improving the air quality of PM_1_, PM_2.5_, PM_10_, and NO_2_ is associated with alleviating frailty progression, but doesn’t find the same positive correlation with O_3_. These findings highlight effective strategies to help slow the frailty progression of older adults, propose interventions that promote positive health outcomes, and reduce countries’ health-care spending [14]. The results of this study provide a valuable insight for China and other developing countries to conduct clean air actions to reduce air pollution and prevent frailty [36,37].

Previous studies have established that air pollution produces adverse effects on health, primarily through mechanisms involving inflammation, oxidative stress, metabolic disturbances, and genetic and epigenetic modifications [38]. Inflammation, in particular, plays a crucial role in the development of various age-related chronic diseases and other negative health outcomes, including depression and dementia [39,40,41,42]. It is reported that there is a positive association between air pollution (especially PM_2.5_) and inflammatory markers, including C-reactive protein (CRP), interleukin 6 (IL-6), tumor necrosis factor-α (TNF-α), and white blood cells [43,44,45,46,47]. Furthermore, elevated levels of inflammatory markers have been associated with accelerated muscle wasting and mass loss, as well as rapid decline in mobility and physical activity among older adults [48,49]. These factors are all key components in defining frailty. Furthermore, air pollution may affect health through systemic oxidation [50,51,52]. The metabolism of organic fraction (i.e., PAHs and nitroPAHs) coated at the surface of PM_2.5_ can produce Reactive Oxygen Species (ROS) [39], and the imbalance between ROS formation and individual antioxidant activity will lead to oxidative stress [53]. Oxidative stress is closely related to inflammation, which can induce inflammation through various pathways [54], thereby affecting frailty. Some studies have demonstrated that air pollution is associated with elevations in HbA1c and insulin resistance [55,56]. Moreover, PM_2.5_ exposure will lead to the development of type II diabetes [57]. Air pollution also has a disruptive effect on homeostasis, leading to increased vulnerability to disease and mortality, and accelerating the decline and deterioration of age-related cellular, tissue, and organ functions [58,59], thereby causing frailty.

Our study possesses several notable strengths. Firstly, our study incorporated data on changes in air pollutant concentrations, enabling us to examine the relationship between air quality improvements and frailty progression. This study is the first to examine the association between improvement of air quality and the progression of frailty. Secondly, there is a lack of studies reporting any potential correlation between PM_1_ and frailty—and our study aims to fill this knowledge gap by presenting new evidence. Thirdly, our study also provides valuable evidence for the population aged 45 years and older, expanding beyond the focus on older adults in previous similar studies. Furthermore, our study specifically investigates the potential protective effect of clean air actions on frailty progression, considering air quality improvement as an important factor. This perspective provides insight into the potential benefits of implementing clean air interventions and policies in preventing or slowing down the progression of frailty. Lastly, this study employs a dynamic assessment approach, allowing for an evaluation of the impact of air quality on frailty progress over time. And this approach enhances the robustness of our results compared to previous studies that primarily focused on the relationship between air pollution and frailty incidence.

However, it is important to acknowledge the limitations of our study. Firstly, the collection of sociodemographic data and self-reported information through questionnaires may introduce recall bias. Additionally, the use of frailty index items based on self-reports may result in an underestimation of the prevalence of certain factors, such as chronic diseases. Secondly, the unavailability of SO_2_ and other pollutants in the CHARLS study hindered the evaluation of the impact of comprehensive pollutants on frailty. Future research should be conducted including a more extensive range of air pollutants when the data are available. Ultimately, although utilizing a random forest model to estimate PM_1_ concentrations, more extensive exposure data still need to be obtained in future study to explore and improve the assessment capability of PM_1_ due to its potential variability in the measurement.

## 5. Conclusions

This nationwide study, conducted in China, examined the relationship between improved air quality in PM_1_, PM_2.5_, PM_10_, NO_2_, and O_3_, and the alleviation of frailty progression among the middle-aged and elderly population. The findings revealed a significant association between enhanced air quality in PM_1_, PM_2.5_, PM_10_, and NO_2_ and the mitigation of frailty progression. However, no significant association was observed between O_3_ reduction and the mitigation of frailty progression in this population. These results illustrated the satisfactory health benefits of the Clean Air Action policy, which reinforced the necessity for continued and enhanced efforts in air pollution quality monitoring and improvment. In addition, as the aging population surges, these findings hold substantial implications for the early strategies of frailty prevention in the general population. It is encouraging to improve air quality via public health measures, as this gives significant benefits in the aspect of healthy aging.

## Figures and Tables

**Table 1:** The characteristics of the study participants at 2011.

Characteristics	Total (n = 12,891)	Without Frailty Progression (n = 6081)	Frailty Progression (n = 6810)	*p*
Age (mean ± SD, year)	58.6 ± 8.8	58.4 ± 8.7	58.7 ± 9.0	0.037
Sex (n, %)				<0.001
Male	6240 (48.4)	3048 (50.1)	3192 (46.9)	
Female	6651 (51.6)	3033 (49.9)	3618 (53.1)	
BMI (mean ± SD, kg/m^2^)	23.62 ± 3.83	23.56 ± 3.77	23.68 ± 3.89	0.096
Residence (n, %)				0.024
Rural	8243 (63.9)	3827 (62.9)	4416 (64.8)	
Urban	4648 (36.1)	2254 (37.1)	2394 (35.2)	
Marital status (n, %)				<0.001
Separated or divorced	1416 (11.0)	678 (11.1)	738 (10.8)	
Married but temporarily separated	835 (6.5)	337 (5.5)	498 (7.3)	
Married and living together	10,640 (82.5)	5066 (83.3)	5574 (81.9)	
Education level (n, %)				0.034
Primary school and below	8664 (67.2)	4018 (66.1)	4646 (68.2)	
Junior high school	2747 (21.3)	1338 (22)	1409 (20.7)	
High school and above	1480 (11.5)	725 (11.9)	755 (11.1)	
Drinking status (n, %)				<0.001
>1 time/month	3342 (25.9)	1668 (27.4)	1674 (24.6)	
<1 time/month	1025 (8.0)	491 (8.1)	534 (7.8)	
Never	8524 (66.1)	3922 (64.5)	4602 (67.6)	
Smoking (n, %)	5140 (39.9)	2502 (41.1)	2638 (38.7)	0.005
Sleeping time (mean ± SD, hours)	6.34 ± 1.82	6.27 ± 1.88	6.39 ± 1.76	<0.001
Napping time (mean ± SD, minutes)	37.48 ± 43.34	37.72 ± 43.71	37.27 ± 43.01	0.554
Insurance (n, %)	12,171 (94.4)	5744 (94.5)	6427 (94.4)	0.839
Social activity (n, %)	6890 (53.4)	3144 (51.7)	3746 (55.0)	<0.001
Cooking fuel (n, %)				0.442
Clean fuel	5515 (42.8)	2580 (42.4)	2935 (43.1)	
Solid fuel	7376 (57.2)	3501 (57.6)	3875 (56.9)	
FI (mean ± SD)	0.22 ± 0.11	0.26 ± 0.11	0.19 ± 0.11	<0.001

BMI: Body mass index, FI: Frailty index.

**Table 2:** Concentration characteristics of air pollution by years.

Pollutant	Total (μg/m^3^)	Without Frailty Progression (μg/m^3^)	Frailty Progression (μg/m^3^)	*p*
2011				
PM_1_	40.02 ± 13.78	40.37 ± 13.61	39.71 ± 13.92	0.006
PM_2.5_	52.35 ± 16.07	52.79 ± 15.90	51.95 ± 16.21	0.003
PM_10_	93.14 ± 28.35	94.15 ± 28.26	92.24 ± 28.40	<0.001
NO_2_	29.37 ± 10.80	29.70 ± 10.78	29.08 ± 10.80	0.001
O_3_	95.08 ± 6.92	95.21 ± 6.92	94.96 ± 6.91	0.045
2015				
PM_1_	38.98 ± 9.80	39.19 ± 9.71	38.80 ± 9.88	0.025
PM_2.5_	53.50 ± 14.21	53.81 ± 14.10	53.22 ± 14.30	0.019
PM_10_	79.16 ± 19.42	79.65 ± 19.28	78.73 ± 19.53	0.008
NO_2_	30.57 ± 8.70	30.82 ± 8.72	30.35 ± 8.68	0.002
O_3_	88.52 ± 7.54	88.59 ± 7.75	88.45 ± 7.35	0.294
∆2011–2015				
∆PM_1_	0.81 (−2.68, 5.30)	1.07 (−2.39, 5.40)	0.79 (−2.82, 5.20)	0.002
∆PM_2.5_	−1.20 (−5.27, 3.21)	−1.03 (−5.14, 3.26)	−1.20 (−5.45, 2.61)	0.008
∆PM_10_	13.40 (6.39, 22.92)	14.51 (6.52, 23.38)	12.27 (5.71, 22.73)	<0.001
∆NO_2_	−1.45 (−4.51, 2.02)	−1.34 (−4.30, 2.12)	−1.53 (−4.73, 1.96)	0.01
∆O_3_	6.52 (3.82, 9.42)	6.52 (3.90, 9.44)	6.52 (3.78, 9.42)	0.309

All data are presented as mean ± SD or median (IQR). PM_1_: particulate matter with an aerodynamic diameter less than 1 µm; PM_2.5_: particulate matter with an aerodynamic diameter less than 2.5 µm; PM_10_: particulate matter with an aerodynamic diameter < 10 µm; NO_2_: nitrogen dioxide; O_3_: ozone.

**Table 3:** Odds ratios (95% CI) for the frailty progression associated with quartiles of air pollution changes.

	Model 1	Model 2	Model 3	Model 4
	OR (95%CI)	OR (95%CI)	OR (95%CI)	OR (95%CI)
∆PM_1_				
Q1	1.00 (ref)	1.00 (ref)	1.00 (ref)	1.00 (ref)
Q2	0.91 (0.83~1.01)	0.91 (0.821~0.999)	0.91 (0.83~1.01)	0.87 (0.78~0.98)
Q3	0.90 (0.82~0.99)	0.89 (0.81~0.99)	0.89 (0.81~0.986)	0.74 (0.66~0.82)
Q4	0.86 (0.78~0.95)	0.85 (0.77~0.94)	0.86 (0.78~0.95)	0.75 (0.68~0.84)
*p* for Trend	0.003	0.002	0.003	<0.001
∆PM_2.5_				
Q1	1.00 (ref)	1.00 (ref)	1.00 (ref)	1.00 (ref)
Q2	0.90 (0.81~0.99)	0.89 (0.81~0.99)	0.88 (0.80~0.98)	0.78 (0.72~0.89)
Q3	0.99 (0.89~1.09)	0.98 (0.89~1.08)	0.98 (0.89~1.09)	0.82 (0.74~0.91)
Q4	0.84 (0.76~0.92)	0.83 (0.75~0.91)	0.83 (0.75~0.91)	0.72 (0.65~0.80)
*p* for Trend	0.005	0.003	0.004	<0.001
∆PM_10_				
Q1	1.00 (ref)	1.00 (ref)	1.00 (ref)	1.00 (ref)
Q2	1.01 (0.92~1.12)	1.01 (0.92~1.12)	1.01 (0.91~1.11)	0.92 (0.83~1.03)
Q3	0.84 (0.76~0.92)	0.83 (0.75~0.91)	0.84 (0.76~0.93)	0.78 (0.71~0.87)
Q4	0.81 (0.74~0.89)	0.80 (0.73~0.88)	0.80 (0.73~0.88)	0.73 (0.66~0.81)
*p* for Trend	<0.001	<0.001	<0.001	<0.001
∆NO_2_				
Q1	1.00 (ref)	1.00 (ref)	1.00 (ref)	1.00 (ref)
Q2	0.89 (0.81~0.98)	0.88 (0.80~0.98)	0.90 (0.81~0.99)	0.89 (0.80~0.99)
Q3	0.84 (0.77~0.93)	0.84 (0.76~0.92)	0.86 (0.77~0.94)	0.86 (0.77~0.95)
Q4	0.88 (0.80~0.97)	0.87 (0.79~0.97)	0.89 (0.80~0.98)	0.79 (0.71~0.88)
*p* for Trend	0.006	0.004	0.012	<0.001
∆O_3_				
Q1	1.00 (ref)	1.00 (ref)	1.00 (ref)	1.00 (ref)
Q2	0.98 (0.89~1.08)	0.98 (0.89~1.09)	0.96 (0.87~1.06)	0.95 (0.86~1.05)
Q3	0.99 (0.89~1.09)	0.99 (0.90~1.09)	0.98 (0.88~1.08)	0.93 (0.84~1.03)
Q4	0.97 (0.88~1.07)	0.97 (0.88~1.06)	0.98 (0.89~1.08)	0.94 (0.85~1.04)
*p* for Trend	0.518	0.515	0.747	0.199

Model 1: unadjusted; Model 2: adjusted for age, sex, and BMI; Model 3: Model 2 + residence, educational level, marital status, smoking, insurance, drinking status, sleeping time per day, social activity; Model 4: Model 3 + additionally adjusted for frailty index at 2011.

## Data Availability

Dataset available on request from the authors.

## Supplementary Materials

The following supporting information can be downloaded at: https://www.mdpi.com/article/10.3390/toxics12070464/s1, Figure S1:The changes of FI in different quartile of pollutant quality improvement from 2011 to 2015 by different pollutant; Figure S2: Correlation of changes in various air pollutants between 2011 and 2015; Figure S3: Associations between the air pollutants decrement and the benefit of frailty progression by BKMR; Figure S4: Effect size of air quality improvement (per IQR increase) on frailty benefit in exploratory subgroup; Table S1: Selection of items in questionnaire of CHARLS to calculate frailty score; Table S2: Adjusted odds ratios (95% CI) for the frailty progression (ΔFI divided by Q3) associated with quartiles of air pollution changes; Table S3: Adjusted odds ratios (95% CI) for the frailty progression associated with air pollution changes for per 1 μg/m^3^ increase.
