# Supplementary material for: Association of Air Quality Improvement and Frailty Progression: A National Study across China
Source: Toxics. 2024 Jun 27;12(7):464. doi: 10.3390/toxics12070464 (PMC11280498; doi:10.3390/toxics12070464)
Supplement: Supplementary file 1 [file toxics-12-00464-s001.zip › toxics-3048008-supplementary.pdf]

Supplementary Materials

Table S1 Selection of items in questionnaire of CHARLS to calculate frailty score*.

Items	Cut-off points
I. Physical limitations (n=18)	
Difficulty with walking 100 Metres.	0=did not have any problems with the activity; 1=some difficulty with the activity or could not do the activity.
Difficulty with controlling urination and defecation.	
Difficulty with getting up from chair after sitting for long periods.	
Difficulty with climbing several flights of stairs without resting.	
Difficulty with stooping, kneeling, or crouching.	
Difficulty with reaching or extending arms above shoulder level.	
Difficulty with lifting or carrying weights over 10 Jin (5kg).	
Difficulty with picking up a small coin from a table.	
Difficulty with dressing.	
Difficulty with bathing or showering.	
Difficulty with eating.	
Difficulty with getting out of bed and walking.	
Difficulty with using the toilet, including getting up or down.	
Difficulty with managing money.	
Difficulty with taking medications.	
Difficulty with shopping for groceries.	
Difficulty with preparing a hot meal.	
Difficulty with cleaning house.	
II. CESD-10 (n=10)	
Felt depressed.	0=Rarely or none of the time; 1= Some or a little of
Felt sleep was restless.	
Felt everything they did was an effort.	

Felt lonely.	the time/ Occasionally or a moderate amount of the time/ Most or all of the time.
Bothered by little things.	
Could not get going.	
Had trouble keeping mind on what is doing.	
Felt fearful.	
Be happy.	0=Most or all of the time; 1=Some or a little of the time/ Occasionally or a moderate amount of the time/ Rarely or none of the time.
Felt hopeful about the future.	
III. Comorbidities (n=13)	
Hypertension.	0=never diagnosed; 1=ever diagnosed.
Diabetes.	
Lung disease.	
Heart problem.	
Stroke.	
Emotional, Nervous, or Psychiatric Problems.	
Arthritis.	
Dyslipidemia.	
Liver disease.	
Kidney disease.	
Stomach/digestive disease.	
Asthma.	
Memory problem.	
IV. History of trauma (n=2)	
Fractured hip.	

Fallen down.	0=never experienced; 1=ever experienced.
V. Cognitive impairment (n=6)	
Cannot answer correct month given.	0=can correctly answer; 1=cannot correctly answer.
Cannot answer correct year given.	
Cannot answer correct day given.	
Numeracy.	
Immediate recall.	0=could correctly recall at least 6 words; 1=could not correctly recall at least 6 words.
Delayed recall.	0=could correctly recall at least 7 words; 1=could not correctly recall at least 7 words.
VI. Others (n=4)	
Hearing problem.	0=never had; 1=ever had.
Eyesight problem.	
Self-reported pain.	0=never troubled with anybody pains; 1=a little/ somewhat/ quite a bit/ very often troubled with body pains.

Self-reported health.	0=very good/ good; 1=fair/ poor/ very poor.

* The frailty index was calculated by summing the number of deficits reported by the respondents and dividing by the total number of possible deficits that were answered. We included 53 health deficits, which met the following criteria: (1) a minimum of 30 deficits in total are included; (2) each deficit is associated with adverse health outcomes; (3) increase in prevalence with age; (4) the prevalence should not be less than 1%; (5) does not saturate.

Table S2

Adjusted odds ratios (95% CI) for the frailty progression (ΔFI divided by Q3) associated with quartiles of air pollution changes.

Pollutant	Case (n, %)	aOR(95%CI)	P	Adjusted for FI at baseline additionally	P
ΔPM_1					
Q1	965 (30.1)	1(Ref)		1(Ref)	
Q2	817 (25.1)	0.80 (0.72~0.90)	<0.001	0.78 (0.69~0.87)	<0.001
Q3	705 (22.4)	0.69 (0.62~0.78)	<0.001	0.61 (0.54~0.69)	<0.001
Q4	736 (22.4)	0.70 (0.63~0.79)	<0.001	0.65 (0.58~0.73)	<0.001
Trend test	3223 (25)	0.89 (0.86~0.92)	<0.001	0.86(0.83~0.89)	<0.001
$\Delta PM_{2.5}$					
Q1	913 (28.8)	1(Ref)		1(Ref)	
Q2	789 (23.9)	0.77 (0.69~0.86)	<0.001	0.72 (0.64~0.81)	<0.001
Q3	814 (25.3)	0.85 (0.76~0.96)	0.006	0.75 (0.67~0.85)	<0.001
Q4	707 (22.1)	0.71 (0.63~0.79)	<0.001	0.65 (0.58~0.73)	<0.001
Trend test	3223 (25)	0.91 (0.88~0.94)	<0.001	0.88 (0.85~0.92)	<0.001
ΔPM_{10}					

Q1	907 (28.4)	1(Ref)		1(Ref)	
Q2	875 (27)	0.91 (0.82~1.02)	0.106	0.86 (0.77~0.96)	0.009
Q3	738 (23)	0.79 (0.71~0.90)	<0.001	0.77 (0.68~0.86)	<0.001
Q4	703 (21.6)	0.71 (0.63~0.79)	<0.001	0.67 (0.60~0.76)	<0.001
Trend test	3223 (25)	0.89 (0.86~0.92)	<0.001	0.88 (0.85~0.91)	<0.001
ΔNO_2					
Q1	909 (28.5)	1(Ref)		1(Ref)	
Q2	840 (26)	0.92 (0.82~1.03)	0.148	0.92 (0.82~1.03)	0.153
Q3	789 (24.3)	0.85 (0.76~0.96)	0.006	0.86 (0.76~0.96)	0.009
Q4	685 (21.3)	0.74 (0.65~0.83)	<0.001	0.69 (0.61~0.77)	<0.001
Trend test	3223 (25)	0.91 (0.87~0.94)	<0.001	0.89 (0.86~0.92)	<0.001
ΔO_3					
Q1	805 (25.1)	1(Ref)		1(Ref)	
Q2	836 (26.1)	1.01 (0.90~1.13)	0.852	1.01 (0.90~1.13)	0.895
Q3	806 (24.8)	0.94 (0.84~1.06)	0.308	0.92(0.81~1.03)	0.136
Q4	776 (24.1)	0.99 (0.88~1.11)	0.870	0.97 (0.86~1.09)	0.582
Trend test	3223 (25.0)	0.99 (0.95~1.03)	0.575	0.98 (0.94~1.02)	0.287

*Adjusted for age, sex, BMI, residence, educational level, marital status, smoking, insurance, drinking status, sleeping time per day and social activity.

Table S3

Adjusted odds ratios (95% CI) for the **frailty progression** associated with air pollution changes for per 1μg/m^3 increase.

**Pollutant**	**Case (n, %)**	**aOR(95%CI)**	**aP**
ΔPM_1	6810 (52.8)	0.978 (0.971~0.985)	<0.001
$\Delta PM_{2.5}$	6810 (52.8)	0.983 (0.977~0.989	<0.001
ΔPM_{10}	6810 (52.8)	0.987 (0.984~0.991)	<0.001
ΔNO_2	6810 (52.8)	0.981 (0.971~0.990)	<0.001
ΔO_3	6810 (52.8)	0.996 (0.989~1.002)	0.1709

*Adjusted for age, sex, BMI, residence, educational level, marital status, smoking, insurance, drinking status, sleeping time per day, social activity and FI at baseline.

Figure. S1 The changes of FI in different quartile of pollutant quality improvement from 2011 to 2015 by different pollutant.

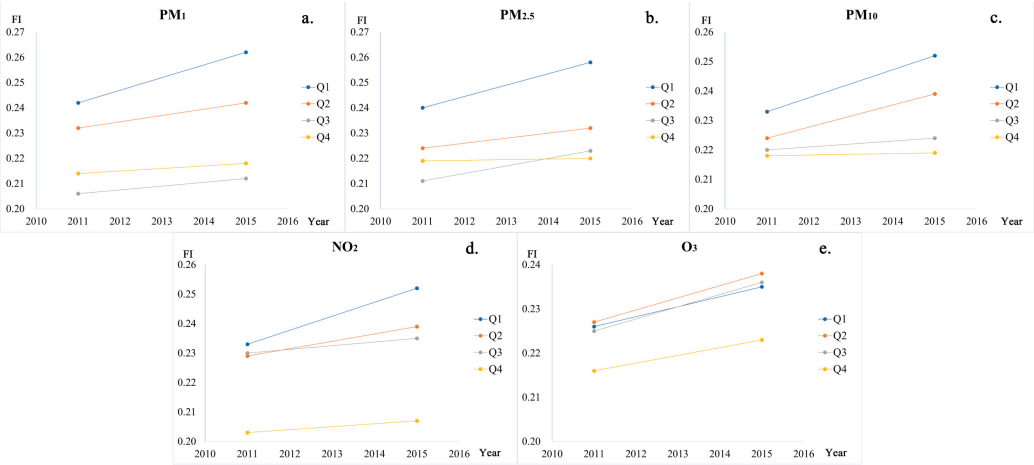

⋆ a. ΔPM_1, b. $\Delta PM_{2.5}$, c. ΔPM_{10}, d. ΔNO_2, e. ΔO_3. Population was divided according to the Quartile of pollutant improvement into four parts. Q1, top 25% of the population; Q2, 25%-50% of the population; 50%-75% of the population; Q4, the last 25% of the population.

Figure.S2 Correlation of changes in various air pollutants between 2011 and 2015.

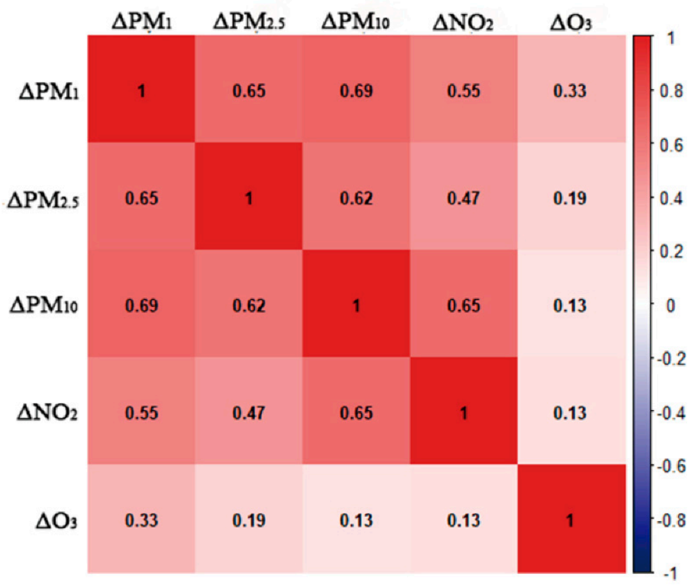

Figure.S3 Associations between the air pollutants decrement and the benefit of frailty progression by BKMR model.

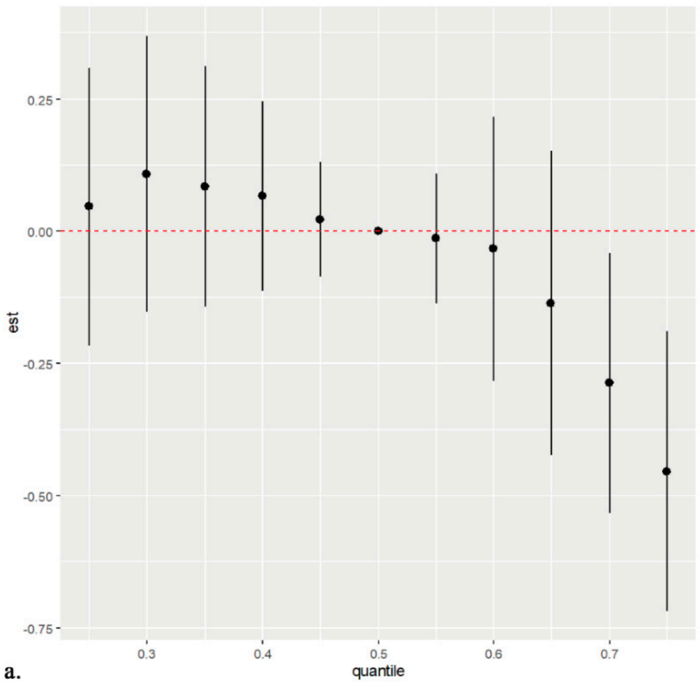

a.

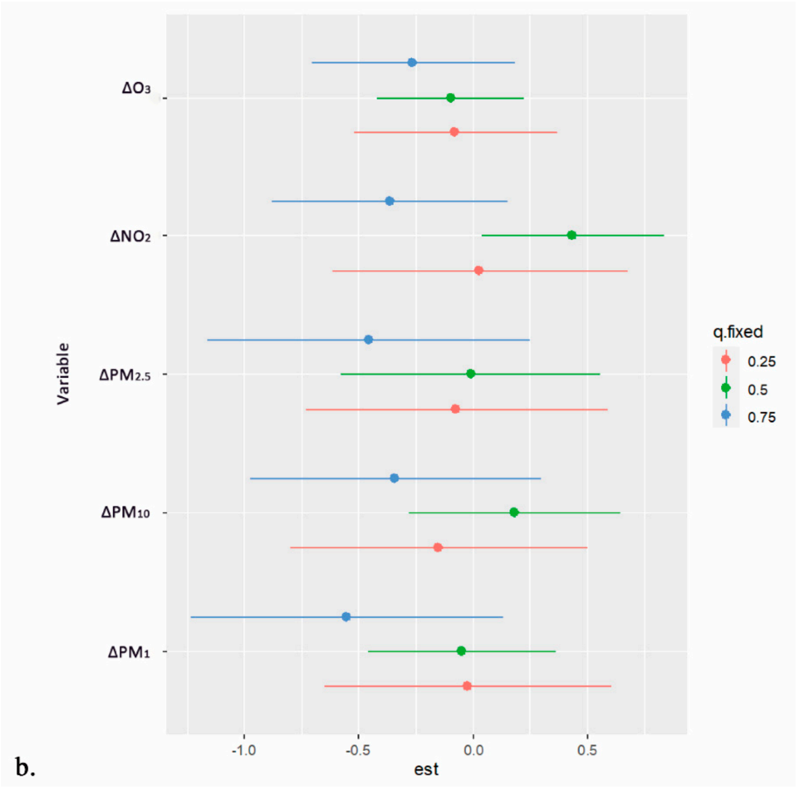

* Models were adjusted for age, sex, and BMI. a. The cumulative effect of the ΔPM_1, $\Delta PM_{2.5}$, ΔPM_{10}, ΔNO_2 and ΔO_3 (estimates and 95% CI). The Y-axis shows the estimated change in risk of ΔFI when the change of all air pollutants were set at particular per- centiles (ranging from 25th to 75th) compared to when the change of all the air pollutants were at their 50th percentile; b. The single-exposure effect (estimates and 95% CI). PM_1: Particulate matter with an aerodynamic diameter <1 μm, $PM_{2.5}$: Particulate matter with an aerodynamic diameter <2.5 μm, PM_{10}: Particulate matter with an aerodynamic diameter <10 μm, NO_2: Nitrogen dioxide, O_3: Ozone.

Figure.S4 Effect size of air quality improvement (per IQR increase) on frailty benefit in exploratory subgroup.

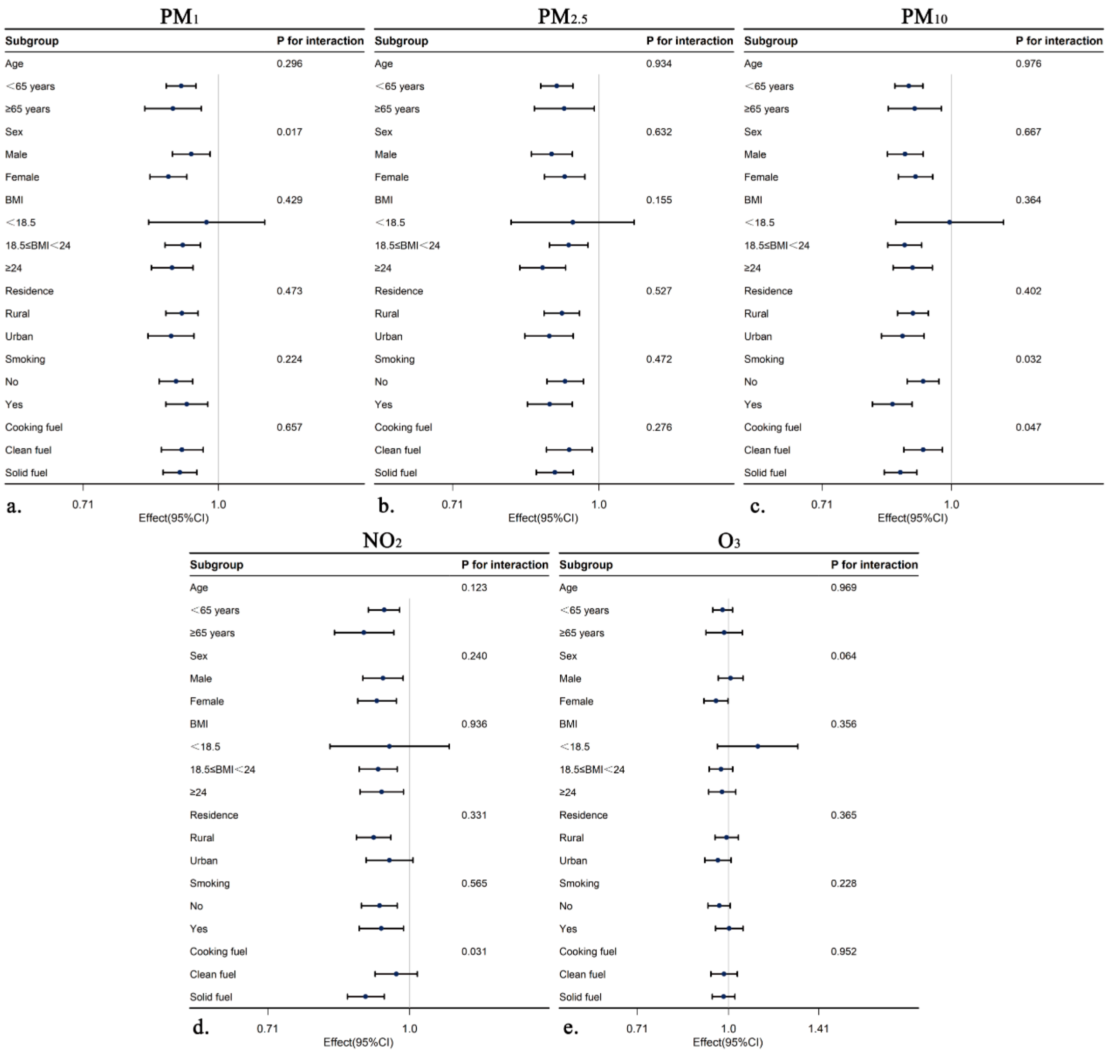

Effect size of air quality improvement (per IQR increase) on frailty benefit in exploratory subgroups based on Model 4. a. PM_1, b. $PM_{2.5}$, c. PM_{10}, d. NO_2, e. O_3. Note. P-value for interaction. PM_1: Particulate matter with an aerodynamic diameter <1 μm, $PM_{2.5}$: Particulate matter with an aerodynamic diameter <2.5 μm, PM_{10}: Particulate matter with an aerodynamic diameter <10 μm, NO_2: Nitrogen dioxide, O_3: Ozone.
